# Hemodynamic Analysis of VenaTech Convertible Vena Cava Filter Using Computational Fluid Dynamics

**DOI:** 10.3389/fbioe.2020.556110

**Published:** 2020-10-30

**Authors:** Jingying Wang, Wen Huang, Yue Zhou, Fangzhou Han, Dong Ke, Chunhian Lee

**Affiliations:** ^1^School of Energy and Power Engineering, Shandong University, Jinan, China; ^2^The First Affiliated Hospital of Chongqing Medical University, Chongqing, China; ^3^School of Aeronautical Science and Engineering, Beihang University, Beijing, China

**Keywords:** inferior vena cava filter, hemodynamics, deep vein thrombosis, computational fluid dynamics, VenaTech convertible filter

## Abstract

The VenaTech convertible filter (VTCF) has been widely used as an inferior vena cava (IVC) filter to prevent fatal pulmonary embolism in patients. However, its hemodynamics that greatly affect the filter efficacy and IVC patency are still unclear. This paper uses computational fluid dynamics with the Carreau model to simulate the non-Newtonian blood flows around the VTCF respectively deployed in the normal, reverse and three converted states in an IVC model. The results show that the prothrombotic stagnation zones are observed downstream from the normal, reverse and small open VTCFs, with the streamwise length is nearly eight times the IVC diameter. The no-slip boundary conditions of the thin-wire VTCF arms lead to the “viscous block” effect. The viscous block accelerates the blood flow by 5–15% inside the IVC and enhances the filter wall shear stress up to nearly 20 times that of the IVC only, which contributes to clot capture and thrombus lysis. The relative flow resistance is defined to evaluate the filter-induced resistance on the IVC blood flow that can be regarded as an index of IVC patency with the filter deployment. The flow resistance of the normal VTCF deployment increases dramatically by more than 60% compared with that of the IVC only and is a little higher (6%) than that of the reverse case. As the VTCF converts to a fully open configuration, the flow resistance gradually decreases to that of no filter. This work shows that even very thin VTCF arms can result in the viscous block effect and may cause significant hemodynamic impacts on clot capture, potential thrombosis and flow impedance inside the IVC. The present study also shows that CFD is a valuable and feasible *in silico* tool for analyzing the IVC filter hemodynamics to complement *in vivo* clinical and *in vitro* experimental studies.

## Introduction

Pulmonary embolism (PE) from deep vein thrombosis (DVT) has become a disease with considerable rates of morbidity and mortality worldwide (Hirsh and Hoak, 1996; Kaufman et al., 2009; Beckman et al., 2010; Zhang et al., 2015; Kearon et al., 2016; Mozaffarian et al., 2016). To prevent PE in patients for whom anticoagulation therapy is ineffective or contraindicated, the inferior vena cava (IVC) filter provides a crucial alternative (Chen Y. et al., 2017; Duffett and Carrier, 2017; Lenchus et al., 2017) that has been in use for more than 40 years and currently appears to be increasing in use (Greenfield et al., 1994; Galanaud et al., 2013; Montgomery and Kaufman, 2016). However, the efficacy and safety of IVC filters remain a matter of dispute, and the corresponding complications are common, such as filter tilting, migration, vein wall penetration, filter-induced thrombosis and recurrent DVT and PE (Rahbar et al., 2011; Deso et al., 2016). In an 8-year follow-up randomized trial conducted by the Prevention du Risque d’Embolie Pulmonaire par Interruption Cave (PREPIC) group, IVC filters were reported to reduce the risk of PE but increase that of DVT with no effect on survival (The PREPIC Study Group, 2005). The subsequent PREPIC2 trial showed that compared with anticoagulant therapy alone, the placement of an IVC filter following short-term anticoagulation did not have any statistically significant benefit in terms of PE recurrence or mortality in patients with acute symptomatic PE (Mismetti et al., 2015). Therefore, efforts still need to be made regarding the assessment and optimization of IVC filters (Magnowski et al., 2017).

At present, there is a consensus that an ideal IVC filter should be non-migratory, non-thrombogenic and if possible, while capturing clots efficiently and maintaining vena cava patency (Leask et al., 2001, 2004; Harlal et al., 2007; Lessne et al., 2016; Montgomery and Kaufman, 2016). Most of the aforementioned concerns depend, to a certain extent, on the hemodynamic characteristics of an IVC filter design (López et al., 2018), which can be directly observed *in vivo* by computed tomography and magnetic resonance imaging (Gaines et al., 2018) or investigated by some *in vitro* experimental techniques, such as photochromic flow visualization (Couch et al., 1997). However, both *in vivo* and *in vitro* measurements do not study certain pathological states or physical models with the use of IVC filters and are always limited to specific patients and the setup ability and by high cost (Swaminathan et al., 2006). Comparatively, the numerical simulation based on *in silico* computational fluid dynamics (CFD) has better potential to be flexible regarding condition settings, reproducible and controllable regarding physical models, and relatively low cost (Swaminathan et al., 2006; Singer et al., 2009; Wang et al., 2019; Shar et al., 2020). Currently, CFD models are widely and successfully employed to simulate the blood flows around different designs of IVC filters (López et al., 2018). The results indicate that the stagnation and/or recirculation zone with low shear stress downstream from the filter possibly traps emboli for potential thrombogenesis (Singer et al., 2009). The simulation data show fairly low levels of turbulence intensity downstream from the IVC filters that would not likely be responsible for platelet activation (Rahbar et al., 2011). CFD can also calculate the total drag exerted by the blood flow on the filter surface that needs to be balanced by the total force exerted by the filter hooks/struts on the IVC wall at the contact locations (López et al., 2018).

**FIGURE 1:**
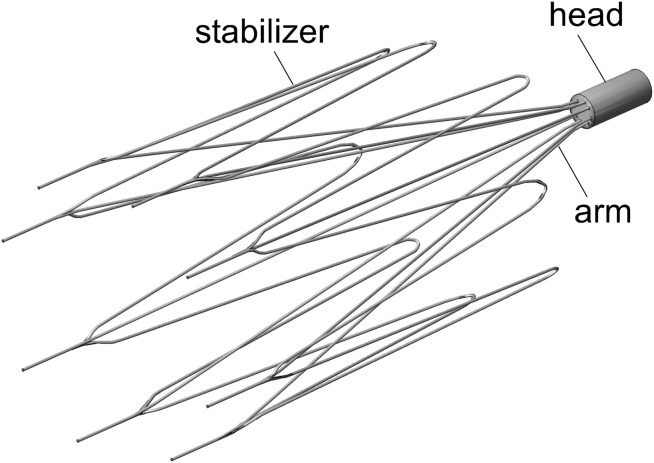
The VenaTech convertible filter model.

The IVC filters on the current market can be generally classified into two categories: temporary (or retrievable) filters and permanent filters (Lessne et al., 2016). The former is clinically recommended to be retrieved out of the patient’s body as soon as the protection from PE is no longer needed. The VenaTech convertible filter (VTCF) (B. Braun, Melsungen, Germany) is a temporary filter made of a cobalt-chromium alloy but uniquely designed to be “converted” based on the VenaTech permanent filter (Le Blanche et al., 2012). The VTCF has eight filtering arms associated with the same number of lateral stabilizers to enhance its non-migratory ability (see Figure 1). The innovative converted design of VTCF is different from ordinary retrieval in that once the period of PE risk has passed, the head at the apex of the filter cone can be removed, unlocking the filter into an open configuration. The VTCF opens its arms opposing the vena cava wall when fully converted, appearing similar to a stent. Although a multicenter clinical trial has demonstrated that the VTCF is a safe temporary filter with high conversion rates and low 6-month complication rates, further research is still necessary to determine the long-term safety and efficacy of the VTCF in both the converted and unconverted states. In the clinical practice, there is some possibility that the VTCF converts unsuccessfully and keeps in a partially open state. A multicenter trial of the VTCF has shown that 19.8 and 5.2% of subjects were considered to be moderately difficult and difficult in the filter conversion, respectively (Hohenwalter et al., 2017). That is, if the accessories were not used, there should be a considerable unconverted rate for VTCF. Therefore, it is of great significance to study the flow characteristics of the partially open state of VTCF. Additionally, some surgeons also have interests in the blood flow dynamics over a VTCF deployed in the reverse state, because some kinds of IVC filters have this similar reversely conical design, for example, the first stage of TrapEase or OptEase (Leask et al., 2004; Lessne et al., 2016).

The objective of this study is to evaluate the hemodynamics of the VTCF deployed in the normal and reverse unconverted states, as well as three converted states with different degrees of opening by using CFD models that simulate the blood flow around the filter and show the distribution of the filter wall shear stress (WSS). The CFD results provide valuable insight into the relationship between the VTCF design and its potential for filter-induced DVT. This paper also specifically investigates the flow resistance of the VTCF, which has not been discussed for the IVC filter in previous experimental or computational studies.

## Materials and Methods

### CFD Models

This work focuses on comparing the flow features of the normal, reverse and different open states of the VTCF deployed in IVC using *in silico* CFD models. Complicated physiological factors, such as the eventual side branches, the IVC deformation and the respiration-induced IVC collapse (Aycock et al., 2016; Tedaldi et al., 2018), are temporarily neglected. Therefore, some primary assumptions need to be made for the CFD simulations. First, due to the low pulsation of blood flow and low pressure measured in the human IVC (Leask et al., 2004), the present IVC model is constructed as a rigid circular tube with a constant blood flow rate. Second, since the VTCF stabilizers are generally incorporated into the IVC wall in the actual treatment (Hohenwalter et al., 2017), the VTCF is assumed to be centrally assembled into the IVC tube with all lateral stabilizers ignored. Finally, blood is a shear-thinning fluid (Cho and Kensey, 1991), the rheological properties of which are taken into account. All the aforementioned assumptions follow the actual clinical practice of the therapy with VTCF and thus can ensure that the present CFD study provides reasonable and reliable flow information around the VTCF in the IVC.

**FIGURE 2:**
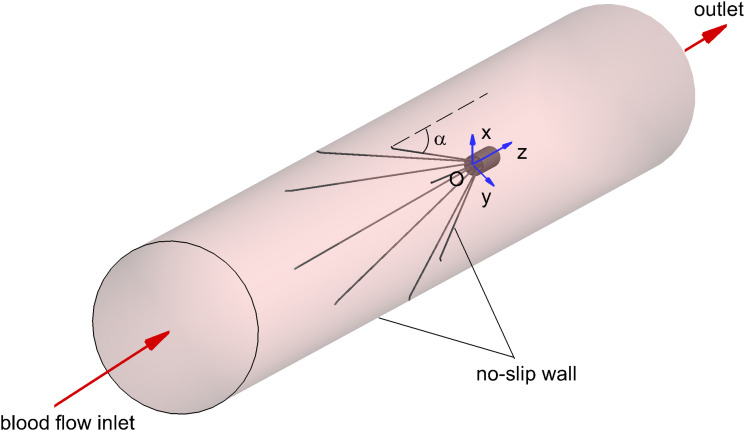
The inclined angle α and CFD boundary conditions.

The three-dimensional model of the VTCF, as shown in Figure 1, is reconstructed on a computer based on the dimensions of a real filter measured using vernier calipers. The open state of the converted VTCF deployed in the IVC is described by the inclined angle, α, between the filter arm and tube wall (see Figure 2).

Considering the low pulsation of the IVC blood flow and pressure, it is reasonable to assume the IVC blood flow is steady (Takizawa et al., 2012; Aycock et al., 2014, 2016; López et al., 2018). In theory, the blood flow over the VTCF is characterized by the viscous, incompressible Navier–Stokes (N–S) equations (Liu et al., 2018) as follows:

(1) $$\left\{ \begin{array}{c} \nabla \cdot \text{V}=0\\ \rho \,\text{V}\cdot \nabla \text{V}=-\nabla p+\nabla \cdot \tau \end{array}\right.$$

where “∇” is the gradient operator, *V* is the velocity vector of the blood flow, ρ is the blood density, and *p* is the static pressure. The viscous stress tensor, τ, is calculated by the following formula (Chen Z. et al., 2017) as:

(2) $$\tau =\mu \,\left[\nabla \text{V}+{\left(\nabla \text{V}\right)}^{T}\right]$$

where μ is the dynamic viscosity of blood and the superscript “T” represents the transposition. Blood is a non-Newtonian fluid, and its viscosity varies with the flow shear rate. Although the use of a Newtonian behavior for blood is widely accepted when simulating blood flows in arterial vascular segments (i.e., the Aorta), the Newtonian blood rheological model could not accurately predict WSS when simulating IVC hemodynamics (Aycock et al., 2016). Therefore, the Carreau model (Cho and Kensey, 1991) is employed in this work to calculate the dynamic viscosity of blood, μ, as follows:

(3) $$\mu =\mu_{\infty }+\left(\mu_{0}-\mu_{\infty }\right)\,{\left[1+{\left(\lambda \,\gamma \right)}^{2}\right]}^{\frac{n-1}{2}}$$

where γ is the local shear rate. The Carreau model has been widely used and validated as adequately accurate for characterizing the non-Newtonian properties of blood in many previous studies (Swaminathan et al., 2006; Fortuny et al., 2015; Aycock et al., 2016; López et al., 2018; Zhou et al., 2018). The model parameters are set as μ_*8*_ = 0.00345 Pa⋅s (also the plasma viscosity), μ_*0*_ = 0.056 Pa⋅s, λ = 3.313 s, and *n* = 0.3568.

The appropriate boundary conditions are necessary for CFD simulations. As shown in Figure 2, the surfaces of the VTCF and IVC tube are both set as no-slip walls where the blood flow speed is zero. One end of the IVC tube is regarded as an inlet, while the other end is considered as the outlet. Flow extensions are added at the inlet and outlet sections, respectively, the lengths of which are both nearly 20 times the IVC diameter to ensure fully developed laminar flow and avoid boundary effects. The Velocity Inlet condition in Fluent is used at the IVC inlet, where the mean flow speed is imposed. The Outflow condition in Fluent is imposed at the outlet, where the flow information is extrapolated from the interior with a zero diffusion flux for all flow variables to be consistent with a fully developed flow assumption.

In the present study, the blood flows for five states of the VTCF deployed in the IVC tube are simulated for comparison including the normal and reverse unconverted cases, as well as three converted cases with different open degrees (small open α = 14°, moderate open α = 10° and large open α = 5°). The IVC diameter, *D*, is taken as 20 mm, the blood density, ρ, is set to 1060 kg/m^3^ and the blood flow rate, *Q*, is 1.134 L/min (the corresponding mean speed of the blood flow in the IVC tube, *V*_*m*_, is 0.06 m/s), which are typical in physiology (Mohan et al., 1995) and set as common parameters for all five cases. The Reynolds number, Re, defined as follows:

(4) $$R\,e=\frac{\rho \,V_{m}\,D}{\mu_{a}}$$

where μ*_*a*_* is the spatially averaged viscosity (Aycock et al., 2016). According to the following CFD results, the spatially averaged viscosity for the normal, reverse and three converted cases is about 0.0097 Pa⋅s, and the corresponding Re is 122, which determines that the blood flow in the present study is laminar.

### Grid Refinement Study

**FIGURE 3:**
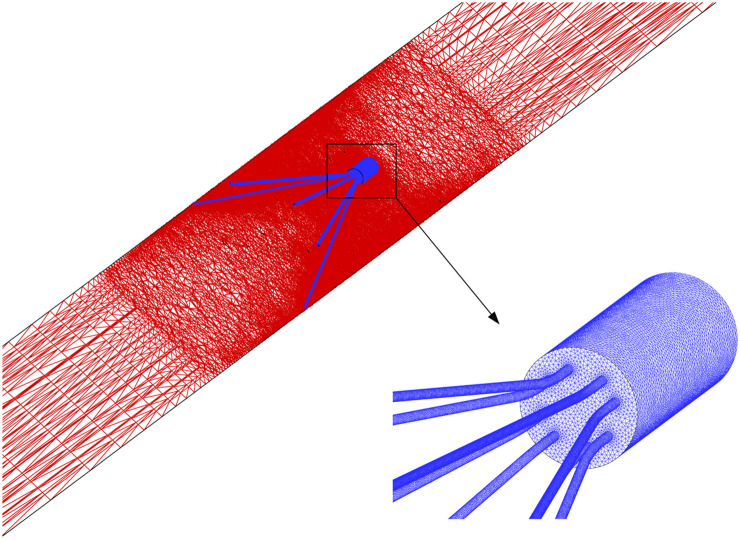
CFD grids surrounding the VTCF.

The blood flow domain of each case is meshed into polyhedral grids for simulation, and the grids surrounding the VTCF are refined adapted to the thin-wire filter arms as shown in Figure 3. A total of six meshes list in Table 1 are used to simulate the normal VTCF deployment case for solution verification with a two-pronged scheme (Roache, 2009; Craven et al., 2018): (i) qualitative evaluation of the effect of mesh resolution on the prediction of blood flow field variables, and (ii) quantitative evaluation of the numerical uncertainty of scalar quantities of interest. The present study focuses on the hemodynamic characteristics of the VTCF deployment in IVC. Therefore, for (i), the velocity profile at *z* = −0.01 m cross-section and the WSS distribution on the upstream side along the filter arm are compared, while, for (ii), the maximum velocity magnitude at *z* = −0.01 m cross-section and the area-averaged filter WSS are considered by calculating both the observed order of convergence, *p*, and the grid convergence index (GCI) (Craven et al., 2018). The area-averaged filter WSS is defined as:

**TABLE 1:** Quantitative evaluation of the CFD uncertainty of maximum velocity magnitude at *z* = −0.01 m cross-section and area-averaged filter WSS for the normal VTCF deployment case predicted using Meshes 1–6.

Mesh	Total number of grid nodes	Maximum velocity magnitude			Area-averaged filter WSS		
		*u*_*max*_ (m/s)	*p*	GCI (%)	WSS_*avg*_ (Pa)	*P*	GCI (%)
1	7.70E + 05	0.087	–	–	1.128	–	–
2	1.02E + 06	0.099	–	–	1.430	–	–
3	1.36E + 06	0.104	3.13	3.92	1.570	2.69	9.70
4	1.81E + 06	0.105	3.10	1.62	1.630	3.00	3.39
5	2.41E + 06	0.107	2.24	1.34	1.656	2.85	1.59
6	3.19E + 06	0.107	3.19	0.32	1.666	3.41	0.46

(5) $${\text{WSS}}_{\text{avg}}=\frac{1}{A}\,\int_{A}\text{WSS}\cdot \text{d}\,A$$

where the integration is performed over the whole filter surface, and *A* is the total area of the filter surface. Figure 4 qualitatively compares the velocity profiles in the *y*-axis direction at *z* = −0.01 m and the WSS distribution along the upstream side along the filter arm obtained using Meshes 1–6. Figure 5 quantitatively shows that both the maximum velocity magnitude and area-averaged filter WSS converge monotonically with the grid refinement toward the exact solution that would be achieved in the limit of infinite mesh resolution. As list in Table 1, Mesh 5 has been capable of yielding the mesh-converged quantitative predictions of the blood flow velocity and filter WSS, with estimated values of CFD uncertainty of only 1.34 and 1.59%, respectively. Therefore, the total number of grid nodes for each case in this study is chosen to be approximately 2.41 million.

**FIGURE 4:**
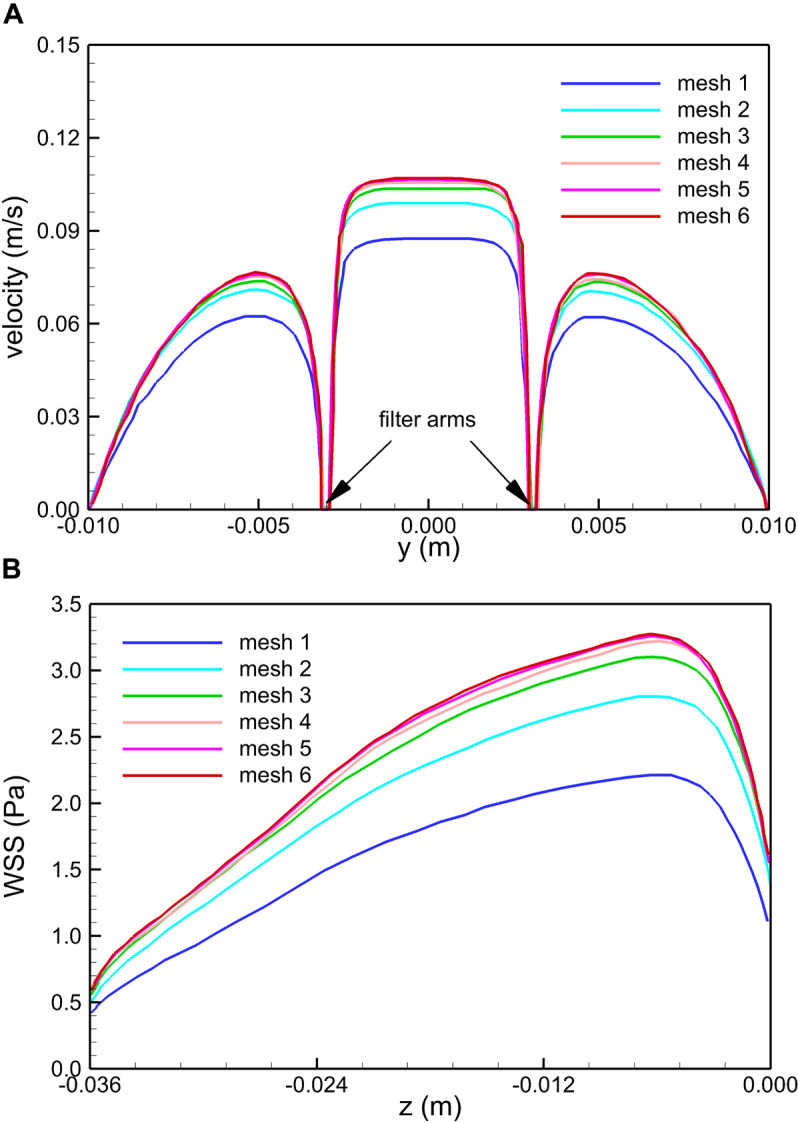
Qualitative comparison of the effect of mesh resolution on the velocity profiles in the *y*-axis direction at *z* = –0.01 m **(A)** and the WSS distribution on the upstream side along the filter arm **(B)** for the normal VTCF deployment case predicted using Meshes 1–6.

**FIGURE 5:**
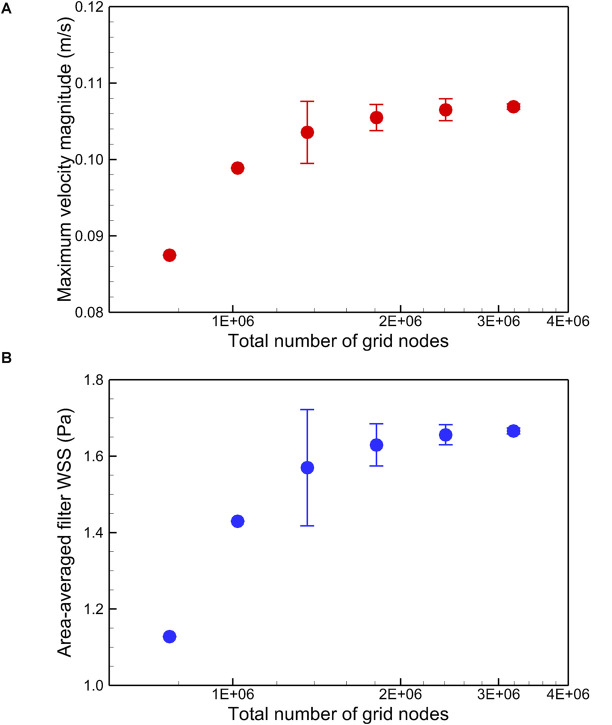
Quantitative comparison of the maximum velocity magnitude at *z* = –0.01 m **(A)** and the area-averaged filter WSS **(B)** for the normal VTCF deployment case predicted using Meshes 1–6. Error bars are plotted for Meshes 3–6 to show the CFD uncertainty quantified by GCI in Table 1.

Computational fluid dynamics solves blood flow eqs 1–3 discretely at each grid node using the algorithm of the semi-implicit method for pressure linked equations (SIMPLE) (Toghraie et al., 2020) in the software FLUENT (ANSYS, Inc., Canonsburg, PA, United States). The simulation process of each case was performed in ten parallel threads on a computer server (Intel i7-8700K 3.70GHz CPU), and each case finished in nearly 2 days by setting the convergent residuals for both the pressure and momentum as 10^–6^.

### Non-newtonian Model Calibration and Comparison Testing

**FIGURE 6:**
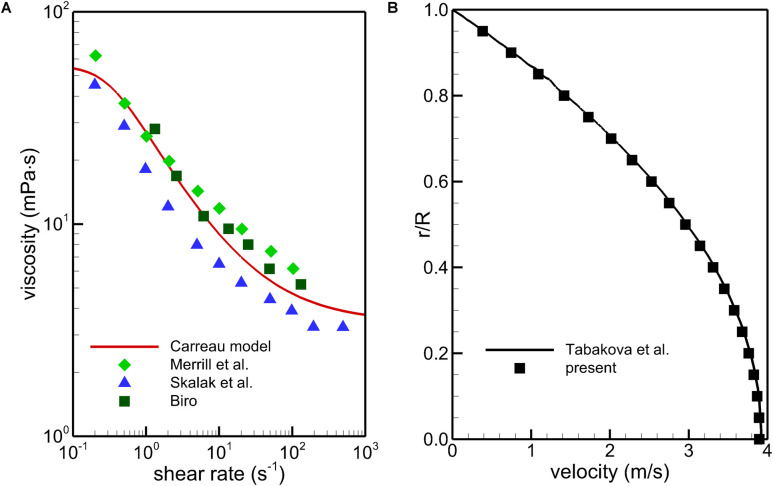
Comparison of the Carreau model with the multi-source experimental data **(A)** and CFD validation using a tube non-Newtonian blood flow case **(B)**.

To obtain quantitatively clinical data of blood flow is a common difficulty for all the IVC filters (Montgomery and Kaufman, 2016). *In vitro* experiment and simulation have become important complementary methods to study the hemodynamic characteristics of the IVC filter (Leask et al., 2001; López et al., 2018). The VTCF is a relatively new IVC filter (Hohenwalter et al., 2017), and there is really lack of applicable clinical and experimental data for verifying the present CFD simulations. Therefore, in this section, there is no experimental validation but only non-Newtonian model calibration and comparison testing to provide the credibility of the present CFD simulation (Salari and Knupp, 2000). Multi-source data (Merrill et al., 1963; Skalak et al., 1981; Biro, 1982) is employed to verify the non-Newtonian Carreau model in section “CFD Models”. FLUENT, the CFD solver in this work has been regarded as a successful tool to simulate blood flows in many studies (Ren et al., 2012; Doost et al., 2016; Chen Z. et al., 2017). A tube non-Newtonian blood flow solution with the Carreau model of Tabakova et al. (2014) is used to examine the simulation accuracy of FLUENT. In the Tabakova et al.’s (2014) case, the tube diameter is 0.0031 m, the blood flow rate is 0.0598 L/s, and the blood density is 1000 kg/m^3^. As shown in Figure 6, the Carreau model under the present parametric settings agrees with the test data well within a wide range of shear rate, and the present CFD results are very consistent with the velocity line of Tabakova et al. (2014). Therefore, the Carreau model can account for the rheological effect of blood and the present CFD solver is reliable for the blood flow simulation.

## Results

### Blood Flow Velocity

**FIGURE 7:**
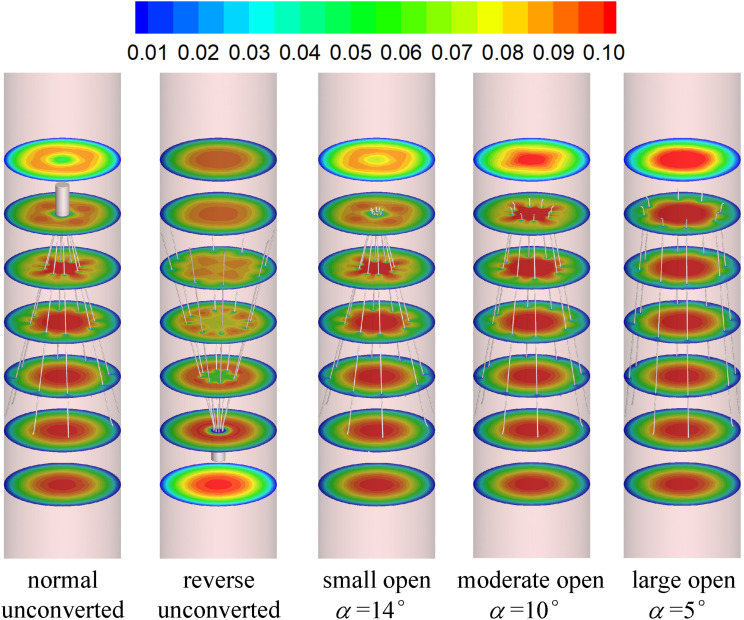
The distributions of blood flow velocity in the cross-sections (unit: m/s).

**FIGURE 8:**
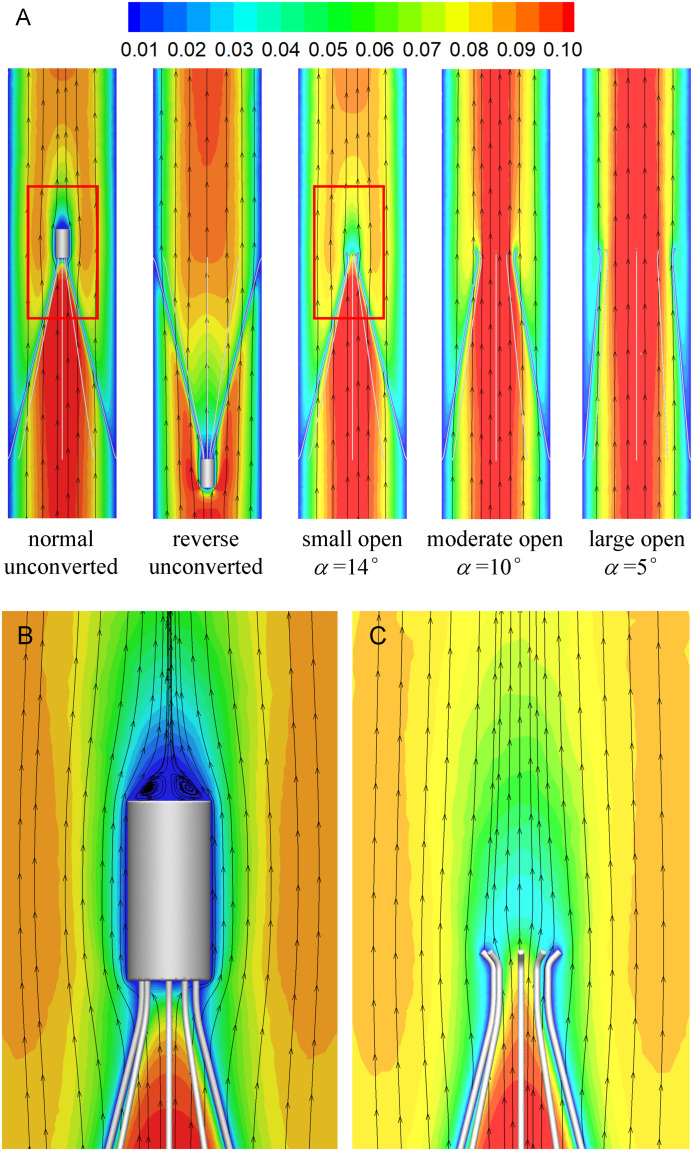
The distributions of blood flow velocity in the axial sections **(A),** zoomed views for the normal **(B),** and small open cases **(C)** (unit: m/s).

**FIGURE 9:**
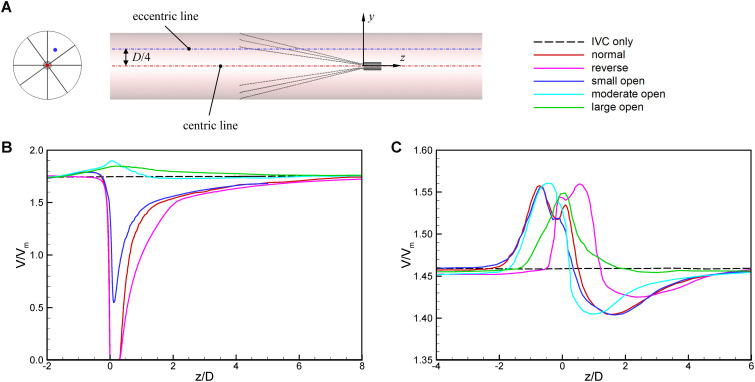
The schematic of IVC centric and eccentric lines **(A),** the variations in blood flow speed along the IVC centric line **(B),** and eccentric line **(C)**. The origin is slightly changed from the bottom center to the top center of the filter head for the reverse case, in order to make the filter head parts of the normal and reverse VTCFs coincide.

Figures 7, 8 show the distributions of blood flow velocity in the cross-sections and axial sections, respectively, for all five states of the VTCF. The high-speed regions are clearly observed inside the filter cone and even between two arbitrary filter arms for the normal unconverted and three converted cases, while a low-speed zone is located in the filter cone for the reverse unconverted case. As demonstrated in Figure 8, obvious stagnation zones can be observed downstream from the normal, reverse and small open filters. As the filter opens more (α = 10° and 5°), the stagnant region disappears. There is also a recirculation region just downstream the filter head (Figure 8B), and once the filter head is removed, this recirculation disappears (Figure 8C). Figure 9 further shows the specific variations of blood flow speed along the IVC centric and eccentric lines, respectively, in which the velocity is non-dimensionalized by the mean flow speed, *V*_*m*_, and the *z*-ordinate is normalized by the IVC diameter, *D*. In Figure 9, for the reverse case, the origin is slightly changed from the bottom center to the top center of the filter head, in order to make the filter head parts of the normal and reverse VTCFs coincide. The accelerated and stagnant effects of the VTCF are seen more clearly from the data lines in Figure 9. Due to the guide function of the thin-wire filter arms, the high-speed blood flows into the IVC central part for the normal VTCF, while the high-speed blood moves toward the IVC wall for the reverse case. Therefore, the blood flow velocity in the stagnation zone just downstream the normal filter head is obviously greater than that downstream of the reverse filter head (see Figure 9B). However, the developed length of the stagnation zone seems insensitive to the filter deployment states, all nearly eight times the IVC diameter.

The acceleration phenomena inside the filter cone and between two filter arms are factually induced by the viscous effect of the filter arms, which are named “viscous block” and discussed in detail later in section “Viscous Block Effect.” For the normal, reverse and small open cases, the stagnation zone spreads downstream from the filter nearly eight times the IVC diameter along the centric line and much longer than that along the eccentric line, approximately five times the IVC diameter. All the figures show a consistent trend between the blood flow around the normal unconverted filter and that of the small open converted case because only the little head of the VTCF is removed for the small open case compared with the normal. As expected, the blood flow surrounding the large open converted VTCF is very similar to that in the IVC only.

### Filter Wall Shear Stress (WSS)

**FIGURE 10:**
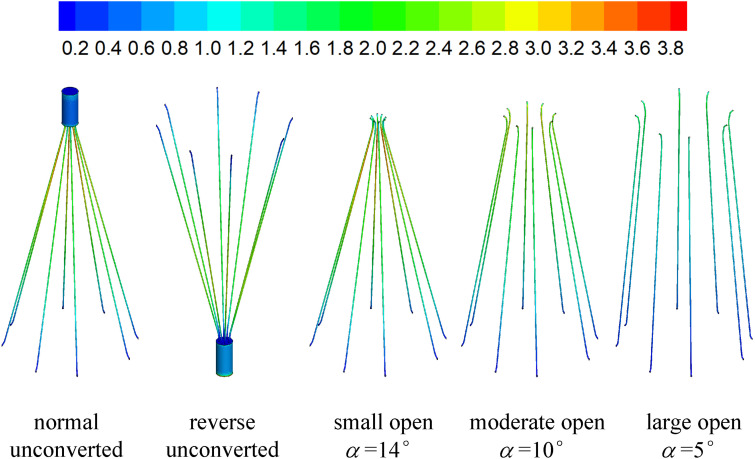
The WSS distributions at the filter surfaces (unit: Pa).

**FIGURE 11:**
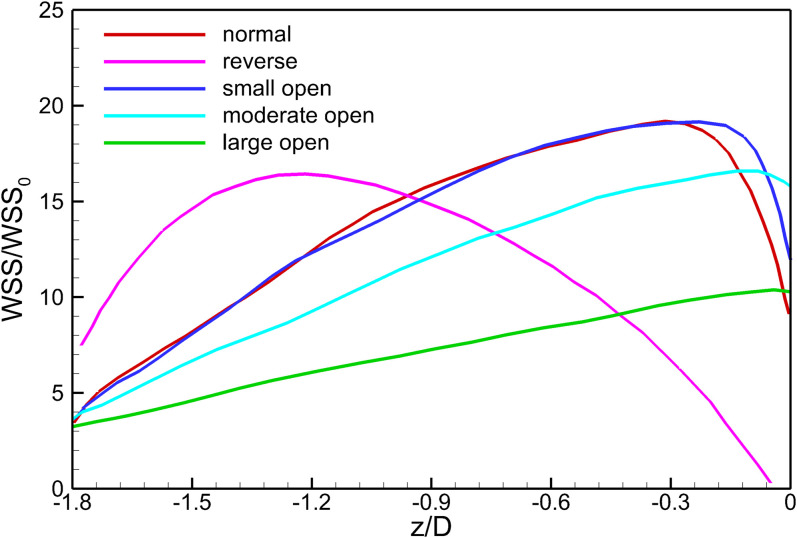
The variations in WSS on the upstream side along the filter arm. WSS_0_ is the IVC wall shear stress value without the filter deployment.

Figure 10 shows the WSS distributions at the surfaces of the VTCF for the normal, reverse and three converted cases. Generally, due to the acceleration of blood flow around the filter arm, the WSS level on the upstream side of the filter arm is much higher than that on the downstream side. Figure 11 show the WSS variations in the upstream side of the filter arm for the five deployment states of the VTCF, where the WSS is non-dimensionalized by the WSS value for the case of IVC only, where WSS_0_ = 0.17 Pa. The lines in Figure 11 further show the variations in WSS on the upstream side along the filter arm. Similar to the former situation of blood flow speed, there is also a consistent tendency between the WSS of the normal unconverted filter and that of the small open filter. For the normal and three open cases, the WSS increases gradually in the flow direction and reaches the maximum near the filter head, while for the reverse VTCF, the WSS increases first and then decreases steadily. As the filter opens, the WSS declines. It is noticeable that the WSS maximum on the upstream side of the filter arm is as high as almost twenty times the WSS value of the IVC only.

## Discussion

A multicenter trial has shown that the VTCF is a safe temporary filter with high conversion rates and low complication rates in a short period (Hohenwalter et al., 2017), but the long-term safety and efficacy of the VTCF in both the converted and unconverted states remain unknown and need more efforts to determine. In the clinical practice, there is some possibility that the VTCF converts unsuccessfully and keeps in a partially open state. Additionally, some surgeons also have interests in the flow dynamics over a VTCF deployed in the reverse state. In the present study, the blood flows for the normal unconverted, reverse unconverted and different converted states of the VTCF deployed in the IVC tube model are simulated by using CFD techniques. The hemodynamics of the VTCF including the flow velocity profiles and filter WSS distributions are discussed in detail, which, to our knowledge, has not been shown in previous experimental or computational studies.

### Stagnation Zone

When the VTCF is placed in the normal, reverse and small open states, a stagnation zone appears with the low speed of blood flow downstream from the filter (see Figures 8, 9), which has also been reported for other IVC filters, such as the Greenfield and TrapEase filters (Swaminathan et al., 2006; Singer et al., 2009). The blood flow stasis is known as a factor of Virchow’s triad involved in intravascular thrombosis (Dickson, 2004) (the other two factors are endothelial damage/abnormality and hypercoagulability of flowing blood). Therefore, the filter-induced region of flow stagnation has been thought to be thrombogenic, which may promote the platelet deposition and fibrin mesh network development for clot formation. The stagnant zone develops to nearly eight times the IVC diameter downstream from the VTCF and that of the reverse case has the lowest speed, as seen in Figure 9B. In reality, the human IVC downstream from the filter is not as long as the present tube model, but the results still, to a certain extent, suggest that the filter disrupts the blood flow inside the IVC greatly and induces hemodynamic potential for DVT.

### Viscous Block Effect

**FIGURE 12:**
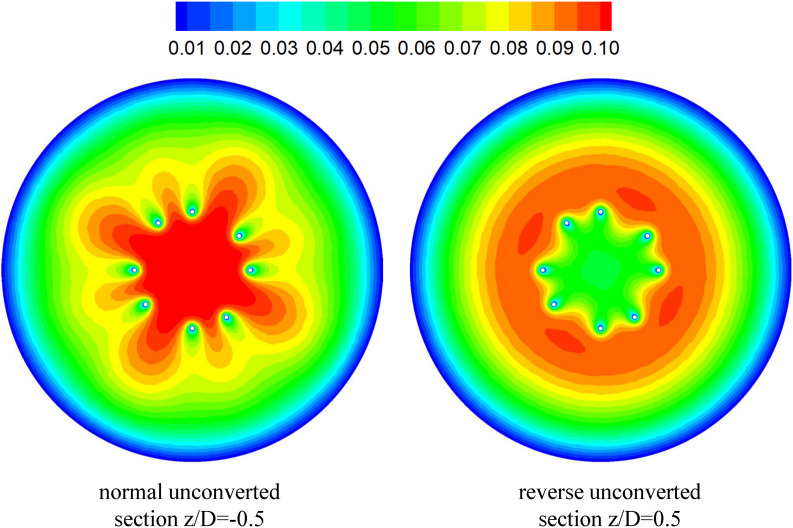
The distribution of blood flow velocity in a certain cross-section (unit: m/s).

The CFD simulations also present a noticeable acceleration effect, with a 5–15% increase in the mean flow speed *V*_*m*_, inside the cone of the VTCF for the normal and three open cases, outside the cone for the reverse case, and between two filter arms for all the cases, which has not been discussed in detail in previous studies. Figure 12 clearly shows the flow acceleration effects in certain cross-sections both for the normal and reverse cases. Due to the viscous no-slip boundary condition in fluid dynamics where the flow speed must be zero at the filter arm surfaces, there is an extensional region of low speed surrounding each filter arm, which leads to a dynamic but not a solid obstruction to impede the blood flow inside the IVC. Simultaneously, the inclination of each filter arm plays a role in guiding the blood flow. Therefore, the normal VTCF cone consisting of eight filter arms acts as a converging duct to speed the inside flow, while the reverse filter cone pushes the blood flow aside and quickly forward to the IVC wall. The interesting acceleration mechanism induced by the viscous no-slip boundary condition is named “viscous block” in this paper. In clinical practice, the viscous block can provide a potential benefit, washing emboli forward to the apex of the filter cone to be captured, but if the VTCF is deployed reversely, the viscous block might result in undesirable clot deposition near the IVC wall. Aycock et al. (2017a, b) has predicted that clots traveling closer to the caval wall are indeed captured at a lower frequency. Although the results of Aycock et al. and the present hypothesis appear consistent, the two physical mechanisms are essentially different. Aycock et al. neglect the influence of the filter struts, and their clot capture efficiency mainly depends on the Segré–Silberberg effect and the spatial structure of the filter struts, while, the present hypothesis is deduced by the viscous block effect of the thin-wire filter arms. Undoubtedly, if the present hypothesis is right, that will further strengthen the clot capture efficiency of Aycock et al. and vice versa. The present complex flow patterns of petals in Figure 12 have also been reported in previous studies for other conical filters, such as Greenfield and Simon Nitinol filters (Swaminathan et al., 2006; Stewart et al., 2008), but the mechanisms were not discussed in depth. Undoubtedly, the viscous block effect can greatly change the IVC blood flow and suggests a significant impact on the filter performance, which disagrees with some previous points of view stating that the filter alone has little effect on the flow (Couch et al., 1997; Aycock et al., 2014). In the future, the viscous block effect should attract more concern in the filter design, and experimental or computational work should be carefully arranged to differentiate the potential effects of various factors.

### Filter Wall Shear Stress

According to Eq. 2, since the shear stress is the product of the blood dynamic viscosity and flow velocity gradient, the acceleration of the viscous block consequently enhances the WSS at the filter surface facing the incoming flow, as shown in Figure 11. Except that of the reverse VTCF, the closer it approaches the head of the filter cone, the higher the WSS is, even as high as 20-fold of the WSS value of the IVC only (WSS_0_ = 0.17 Pa). There is a WSS peak at the upstream surface of the reverse VTCF arm, with a value of nearly sixteen times WSS_0_. The increasing WSS causes two possibilities: one is the benefit that higher WSS tends to favor clot dissolution (Turitto and Hall, 1998); the other is that high shear rate can activate platelet aggregation, which might occur on the VTCF arms and spread into the downstream stagnant zone with the potential for thrombosis formation or propagation (Huang and Hellums, 1993). Therefore, more research on the VTCF is necessary to confirm the aforementioned two effects.

### Flow Resistance

An ideal filter is required to be non-thrombogenic while providing high clot capture efficacy without impeding IVC blood flow. To evaluate the IVC patency for the VTCF deployment, the present study proposes a concept of relative flow resistance (RF) defined as follows:

(6) $$\text{RF}=\frac{\Delta \,P-\Delta \,P_{0}}{\Delta \,P_{0}}$$

where Δ*P* is the pressure drop in the flow direction measured from a segment of IVC tube containing the VTCF, for example, from *z* = −50 mm to *z* = 10 mm, and Δ*P*_0_ is the pressure drop along a segment of the IVC only with the same length as that for Δ*P*. Actually, in the steady blood flow, Δ*P* is a force balanced by the sum of the flow drag exerted by the filter and the viscous friction induced by the IVC wall, while Δ*P*_0_ is only an indicative of the latter. Therefore, in fluid mechanics, RF can be used to evaluate the filter-induced resistance on the IVC blood flow or considered to be an index of IVC patency for filter deployment. The smaller RF is, the better the IVC patency. Table 2 lists the pressure drops and the relative flow resistances for all five states of the VTCF deployed in the IVC. The pressure drop for the IVC only is also Δ*P*_0_. The normal VTCF deployment causes a dramatic RF increase by more than 60% compared with that of the IVC alone. It is interesting that the RF of the reverse filter is lower than that of the normal filter, which occurs because when the head of the filter cone faces the incoming blood flow, it reduces the flow drag in fluid mechanics. Despite the low RF, the reverse state traps emboli near the IVC wall, which leads to a downstream prothrombotic region of stagnant and/or recirculating flow with low shear stress (Stewart et al., 2008; Singer et al., 2009) that is undesirable in clinical practice. As the VTCF gradually opens, naturally, the filter RF decreases steadily. When the VTCF is fully converted to a stent, the RF, in theory, should be equal to that of the IVC only. Table 2 also shows that the pressure drops for all cases are very small (<0.041 mmHg). In fact, given the relatively large diameter of the vena cava and neglecting gravitational effects, the absolute pressure drop over the unoccluded infrarenal IVC is generally extremely small (<0.1 mmHg) (Aycock et al., 2014). Thus, there will a real problem that even a 100% RF value may not be clinically relevant and misleading to some extent. Therefore, the present RF metric should be evaluated cautiously in the clinic.

**TABLE 2:** Pressure drops (Δ*P*) and relative flow resistances (RF) for all cases.

Case	Δ *P* (mmHg)	RF (%)

IVC only	0.0249	–
Normal	0.0408	63.9
Reverse	0.0393	58.0
Small open	0.0401	61.1
Moderate open	0.0374	50.5
Large open	0.0329	32.2

### Study Limitations

There are still certain limitations in the present CFD models. Considering the relatively low pulsatility of the IVC blood flow with low pressure, the IVC tube is assumed to be rigid, and the flow rate is constant, which is adequate for determining the main hemodynamic characteristics herein but only provides the first insight. However, the more actual factors including the eventual side branches, the IVC deformation and the respiration-induced IVC collapse that affect the simulation results of IVC blood flow greatly should be considered in the next step (Aycock et al., 2016; Tedaldi et al., 2018). Additionally, although the mean boundary conditions are used and acceptable for the present study, these conditions deviate from the reality (Craven et al., 2018) and should be further improved. Another limitation is that this study only discusses the state of the VTCF alone with no trapped embolus, which would have a profound effect on the blood flow. In section “Flow Resistance,” RF is proposed to evaluate the filter flow resistance or IVC patency, the concept of which has the clear meaning in physics and well suitable for the present CFD study. However, given the real pressure drop over the IVC segment is very small (<0.1 mmHg), even a very high RF value (e.g., 100%) may not be clinically relevant and misleading to some extent. Therefore, the present RF really should be used cautiously, especially in the clinic and is expected to be improved in the future.

## Conclusion

The blood flows for the normal unconverted, reverse unconverted and three converted states of the VTCF deployed in the IVC are simulated using CFD models for comparison to investigate the hemodynamics of the VTCF including the flow velocity profiles, the filter WSS distributions, and the flow resistances, which have not been discussed in previous experimental or computational studies. The main findings are as follows:

1. There is a stagnation zone with the low speed of the blood flow downstream from the VTCF deployed in the normal, reverse, or small open state, the length of which is nearly 8 times the IVC diameter. This flow-induced stagnation has been thought to be prothrombotic.
2. Even very thin VTCF arms can induce the viscous block effect, which can accelerate the blood flow speed inside the IVC by 5–15% and enhance the filter WSS up to nearly 20 times the WSS value of the IVC only. The viscous block effect may cause significant hemodynamic impacts on clot capture, vein thrombosis and flow impedance.
3. The normal VTCF deployment causes a dramatic flow resistance increase of more than 60% compared with that of the IVC only. As the VTCF converts to a fully open state, the flow resistance of the filter decreases steadily to that of the IVC only.
4. The present study shows that CFD is a valuable and feasible tool for characterizing the blood flow around the IVC filter and calculating the filter resistance in the IVC to complement *in vivo* clinical and *in vitro* experimental studies.

## Data Availability Statement

The original contributions presented in the study are included in the article/supplementary material, further inquiries can be directed to the corresponding author.

## Author Contributions

WH, JW, and CL designed the study. DK constructed the VTCF model. JW and YZ performed the grid refinement study. JW and FH performed the CFD simulations. WH, JW, and DK analyzed the CFD data. JW, WH, FH, and DK wrote the manuscript. All authors contributed to the article and approved the submitted version.

## Conflict of Interest

The authors declare that the research was conducted in the absence of any commercial or financial relationships that could be construed as a potential conflict of interest.
